# A Comparative Study on the Application of Robotic Hair Restoration Technology Versus Traditional Follicular Unit Excision in Male Androgenetic Alopecia

**DOI:** 10.1111/jocd.16554

**Published:** 2024-09-19

**Authors:** Yifei Zhu, Kai Yang, Jui‐Ming Lin, Chunya Ni, Yue Zhang, Zheng Li, Qingmei Liu, Yinghui Zhou, Jinran Lin, Wenyu Wu

**Affiliations:** ^1^ Department of Dermatology, Shanghai Institute of Dermatology, Huashan Hospital Fudan University Shanghai P.R. China; ^2^ Department of Dermatology Jing'an District Central Hospital Shanghai P.R. China; ^3^ Academy for Engineering and Technology Fudan University Shanghai P.R. China

**Keywords:** alopecia, ARTAS, follicles, FUE, hair restoration, robotic surgery

## Abstract

**Background:**

The robotic hair transplant technology, ARTAS, has a series of fine mechanical structure and an intelligent recognition system that allows it to independently select hair follicular units (FUs) and effectively harvest hair. After entering China in 2016, ARTAS has attracted the attention of hair transplant surgeons and hair loss patients given its advantages in a short learning curve and simple operation.

**Objective:**

To compare the efficacy and safety between the ARTAS system and follicular unit excision (FUE) in the treatment of male androgenetic alopecia (AGA) and to further promote the improvement and upgrading of ARTAS technology concerning hair transplantation.

**Methods:**

Thirteen Chinese male patients with Norwood–Hamilton II–IV AGA aged 25–35 years were enrolled in this study. The donor site of each patient was randomly divided into left and right regions, receiving ARTAS on one side and FUE on the other. Yield, transection, and discard rates of hair FUs from both sides were compared. Safety of the procedures in whole, as well as follow‐up results were investigated and evaluated.

**Results:**

The total yield rate on the ARTAS side was lower than on the FUE side (82.05% vs. 90.03%, *p* > 0.05); the total discard rate on the ARTAS side was higher than on the FUE side (10.71% vs. 5.46%, *p* < 0.05); the total transection rate on the ARTAS side was lower than on the FUE side (13.17% vs. 13.96%, *p* > 0.05). No significant difference was found in patient satisfaction (efficacy), and no side effects or complications were detected during or after all surgeries.

**Conclusion:**

The current iteration of Robotic hair transplant technology is effective and safe, and can be recommended for AGA hair transplantation surgery.

## 1 Introduction

Androgenic alopecia (AGA) is a common dermatological disease, affecting 60%–70% of the adult population globally [1]. By the age of 50, up to 40% of women and 50% of men may suffer from AGA to different extent [2]. AGA manifests as receding hairline in the frontotemporal region and thin and soft hair in the parietal region. Onset normally occurs at marriageable age when appearance is more valued, thus increasing number of patients looking to address their AGA has seen significant increase in today's society. At present, the non‐surgical treatments of AGA include oral finasteride, topical minoxidil, low‐level light therapy, and platelet‐rich plasma injection [3]. Although these methods are effective in alleviating hair loss, they are unable to return ideal results in scalp areas where hair follicles have already atrophied, with the forehead and parietal areas being the most susceptible.

Surgical treatment for AGA patients in areas where hair follicles have closed remains the only option, with approaches such as unshaven FUE hair transplant (UFUE) able to achieve better appearance immediately after surgery. Hair transplantation is based on the donor dominance theory [4]. When hair follicle units (FUs) are extracted from the occipital scalp and transplanted to the forehead scalp, their genetic characteristics will be maintained, that is, they are not susceptible to the influence of androgens that leads to hair follicle miniaturization. In the long run, the surgical results are satisfactory. The hair follicle extraction technology used for hair transplantation in the early stage is the follicular unit transplantation (FUT), which has caused problems such as relatively large wounds, slow wound healing, and apparent linear scar. Today, it has been predominantly replaced by follicular unit excision (FUE) that carries advantages of quick recovery and smaller punctate scars [5].

In 2011, the Food and Drug Administration (FDA) approved the first robotic system—ARTAS for hair transplantation. Through technical innovation, the ARTAS robotic hair transplantation system combines modules such as a double needle system, a multi‐axis mechanical arm, a high‐precision imaging system, and an artificial intelligence recognition system to independently select FUs and harvest hair follicles with minimum loss [6]. In 2016, ARTAS entered China and attracted the attention of hair transplant surgeons and hair loss patients because of its short learning curve, simple operation, and other advantages. To understand the advantages and disadvantages of ARTAS technology compared with traditional FUE, and to further promote the improvement and upgrading of ARTAS technology to better service hair transplantation, this investigation was conducted.

## 2 Materials and Methods

This single‐center, prospective, randomized, single‐blinded, controlled, split‐scalp pilot study was conducted at Huashan Hospital, Fudan University between April 2020 and June 2021 (identifier: ChiCTR2000030220). The study protocol was approved by the Huashan Institutional Review Board and adhered to the Declaration of Helsinki and Good Clinical Practice guidelines. All participants provided written informed consent.

Thirteen male patients aged 25–35 with Norwood‐Hamilton II–IV AGA were enrolled. Norwood 2–4 stages were chosen to ensure a consistent comparison across a similar severity range. Details of inclusion and exclusion criteria are:

### 2.1 Inclusion Criteria

1. Patients diagnosed with androgenetic alopecia (AGA), with a Norwood–Hamilton classification of II–IV.
2. Male patients aged between 25 and 35 years, who have been in a stable hair loss phase for more than 1 year.
3. Patients who agree to cut their hair short (1–1.4 mm) and undergo scalp micropigmentation for localization marks.
4. Patients who fully understand the purpose and content of this study, sign the informed consent form, and are willing to undergo both robotic hair transplantation and traditional FUE hair transplantation surgery.

### 2.2 Exclusion Criteria

1. Patients with telogen effluvium, scarring alopecia, alopecia areata, or hair loss caused by physiological factors, medication, physical or chemical factors (including trauma), or other diseases.
2. Patients with any disease or symptoms that may affect the evaluation of efficacy (systemic diseases, keloid tendency, immunodeficiency, or other scalp skin diseases) which could hinder accurate hair counting.
3. Patients who have received systematic hair loss treatment or undergone hair transplantation surgery in the past 6 months.

The number of grafts was limited to maintain uniformity and control within this pilot study. The donor site of each patient was randomly divided into left and right parts using a computer‐generated randomization sequence, with ARTAS employed on one side and FUE on the other. The surgeries were performed by experienced surgeons with over 10 years of experience, several academic publications on alopecia and have conducted over 1000 hair surgeries. They used the FUE Hair Follicle Drilling Machine PK‐7000 equipped with standard serrated punches. For very fine hairs, punches with inner diameters of 0.6 mm were used. FU with hairs directed outwards under the skin were extracted using 1.0 mm punch. The ARTAS system has software version 4.8.2 installed, and used 1.0 mm punch throughout. ARTAS was always performed first and FUE second. Adjustments were made based on real‐time analysis of hair follicle characteristics, and the same surgeon conducted all surgeries to eliminate inter‐operator variability. Each dissection area was approximately 3 × 2 cm^2^, with parameters such as distribution direction, angle, and rotations‐per‐minute set automatically by the robotic system. Typically, 6–10 skin tensioner applications were required to harvest 1000 FU grafts on both sides.

Hair follicles were collected from three grids (upper center, lower center, left lateral) to represent the harvest performance on different scalp regions. The yield rate, transection rate, and discard rate of hair FUs from both ARTAS and FUE sides were compared, with FUs classified according to the number of hair shafts and the area of source. The relationship between the discard rate and the transection rate of different types of FU was evaluated. The literature reports the following distributions: one‐hair FUs (20%–30%), two‐hair FUs (50%–60%), and three‐hair FUs (10%–20%) [17]. Side effects and adverse reactions during and after the operation were also investigated.

### 2.3 Statistical Analysis

The data was analyzed with SPSS Statistics Version 26 and Graphpad Prism Version 9 was used for statistical analysis. The yield, discard rate, and transection rate of the two extraction methods were compared using paired t test. The test level *α* was taken as 0.05, and *p* < 0.05 indicated that the difference was statistically significant.

## 3 Results

### 3.1 Basic Information of Subjects

Demographics and clinical data of each patient are listed in Table 1. Thirteen male AGA patients with mean age 29 years were enrolled.

**TABLE 1:** Demographics and clinical data of each patient.

Patient	Age, years	Current duration, years	Stage scaled by Hamilton–Norwood
1	23	2	III
2	25	3	II
3	33	8	IV
4	27	5	III
5	26	6	III
6	35	5	III
7	30	10	IV A
8	26	6	III
9	31	6	III
10	35	8	III
11	31	7	III
12	25	2	III
13	24	1	II

### 3.2 Comparison Between ARTAS and FUE

In the procedure of hair follicle extraction, several factors can influence the efficiency of the extraction. These factors include the discarding of hair follicles, hair transection, and unsuccessful extraction. Therefore, three indicators, namely, the discard rate, the transection rate, and the yield rate, were introduced to evaluate the efficiency of hair extraction. The two indicators, the discard rate and transection rate, evaluate the damage of the extracted hair follicle and the hair shaft.

However, not all operations can successfully extract hair follicles, and extractions resulting in hair follicles that cannot be further used were deemed invalid extractions. The proportion of the number of hair follicles effectively extracted throughout the full extraction process was recorded as the yield rate. Thus, invalid extractions and discards reduce yield rate.

**FIGURE 1:**
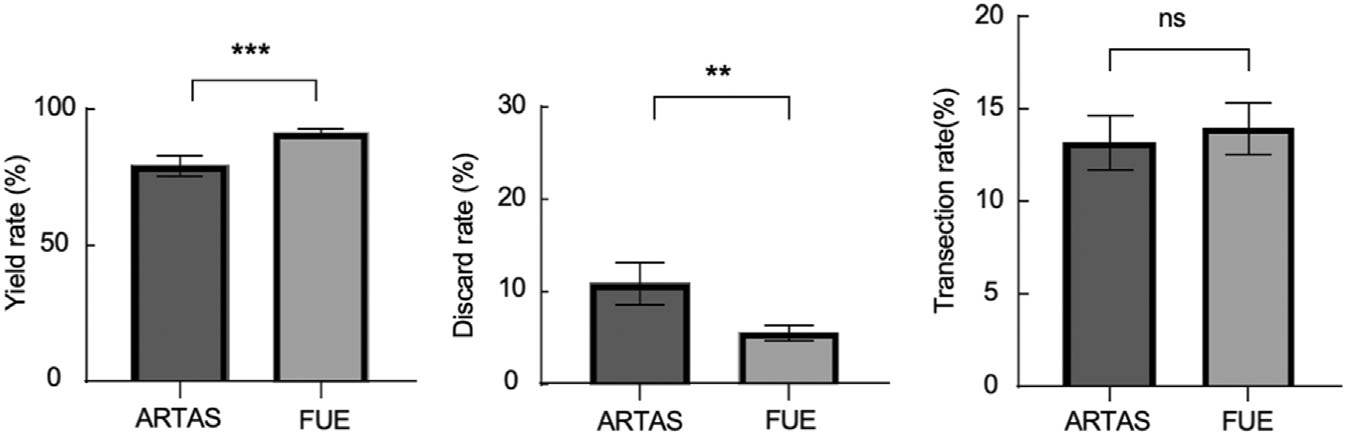
Total yield rate, total discard rate, and total transection rate. “*” stands for statistical difference (*p* < 0.05), while ns stands for no statistical difference. **p < 0.01; ***p < 0.001.

The yield rate, discard rate, and transection rate of each subject are shown in Tables 2, 3, 4. After calculating and statistically comparing total yield rate, discard rate, and transection rate, Figure 1 was obtained. The total discard rate of ARTAS was 10.71%, significantly higher than 5.46% on the FUE side. The total transection rate of ARTAS side was lower than that of FUE side, 13.17% and 13.96%, respectively, with no statistical significance.

**TABLE 2:** Yield rate of each patient.

	Punch		FUs extracted		Yield rate	
	ARTAS	FUE	ARTAS	FUE	ARTAS	FUE
1	955	737	683	670	71.52%	90.91%
2	1118	827	897	586	80.23%	70.86%
3	998	793	900	761	90.18%	95.96%
4	1049	857	826	821	78.74%	95.80%
5	743	572	655	527	88.16%	92.13%
6	652	472	547	459	83.90%	97.25%
7	782	782	555	665	70.97%	85.04%
8	845	845	681	730	80.59%	86.39%
9	1066	1066	861	987	80.77%	92.59%
10	1026	855	757	763	73.78%	89.24%
11	961	961	877	921	91.26%	95.84%
12	860	860	699	711	81.28%	82.67%
13	712	492	678	471	95.22%	95.73%
Average	905	778	740	698	82.05%^a^	90.03%^a^

^a^ 82.05%, 90.03% were averages of each cases.

**TABLE 3:** Discard rate of each patient.

	FUs extracted		FUs discarded		Discard rate	
	ARTAS	FUE	ARTAS	FUE	ARTAS	FUE
1	834	730	151	60	18.11%	8.22%
2	952	613	55	27	5.78%	4.40%
3	998	778	17	17	1.85%	2.19%
4	868	840	42	19	4.84%	2.26%
5	725	573	70	46	9.66%	8.03%
6	612	466	65	7	10.62%	1.50%
7	555	744	151	79	27.21%	10.62%
8	751	780	70	50	9.32%	6.41%
9	1008	1056	147	69	14.58%	6.53%
10	943	846	186	83	19.72%	9.81%
11	941	965	64	44	6.80%	4.56%
12	760	743	61	32	8.03%	4.31%
13	697	481	19	10	2.73%	2.08%
Average	819	740	84	42	10.71%^a^	5.46%^a^

^a^ ARTAS averaged 10.71% discard rate, while FUE averaged 5.46%.

**TABLE 4:** Transection rate of each patient.

	Total hairs extracted		Total hairs transected		Transection rate	
	ARTAS	FUE	ARTAS	FUE	ARTAS	FUE
1	1472	1737	278	322	18.89%	18.54%
2	1687	1689	123	178	7.29%	10.54%
3	1972	2034	99	249	5.02%	12.24%
4	1866	2290	159	305	8.52%	13.32%
5	1593	1403	301	253	18.90%	18.03%
6	1258	1171	196	72	15.58%	6.15%
7	1114	1830	253	463	22.71%	25.30%
8	1606	2023	200	304	12.45%	15.03%
9	1677	2353	209	268	12.46%	11.39%
10	1818	1914	292	337	16.06%	17.61%
11	1789	2117	122	160	6.82%	7.56%
12	1307	1632	172	226	13.16%	13.85%
13	1647	1343	220	160	13.36%	11.91%
Average	1601	1810	202	254	13.17%^a^	13.96%^a^

^a^ ARTAS averaged 13.17% transection rate, while FUE averaged 13.96%.

Normalization was performed for discard rate and transection rate, where results can be found in Table 5. Effects from greater portions of hair count in follicles extracted were lessened, and effects from lesser portions of hair count in follicles were made greater to achieve balance.

**TABLE 5:** Count and percentage of each type of follicular unit.

Patient no.	ARTAS					FUE				
	Ratio			Discard rate^a^	Dissection rate^a^	Ratio			Discard rate^a^	Dissection rate^a^
	1‐hair	2‐hair	3‐hair			1‐hair	2‐hair	3‐hair		
1	37.82%	50.30%	11.88%	1.24	2.18	7.03%	52.46%	40.51%	6.76	7.32
2	19.38%	45.04%	35.57%	1.43	1.71	6.82%	31.63%	61.55%	6.30	6.63
3	19.68%	54.58%	25.74%	0.43	0.75	4.88%	40.45%	54.67%	4.17	4.55
4	23.80%	48.80%	27.39%	0.71	1.08	4.90%	31.92%	63.18%	5.06	5.48
5	18.57%	54.24%	27.19%	1.51	2.21	9.72%	45.50%	44.77%	5.59	6.18
6	24.21%	48.92%	26.87%	1.52	1.99	5.62%	44.72%	49.66%	3.58	3.77
7	37.76%	34.35%	27.89%	2.13	2.56	8.40%	38.60%	52.99%	6.45	7.13
8	19.75%	56.33%	23.92%	1.94	2.42	5.61%	45.14%	49.25%	10.60	11.04
9	44.82%	44.92%	10.26%	0.91	1.48	12.32%	57.23%	30.46%	2.98	3.38
10	25.19%	59.06%	15.76%	2.69	3.26	9.29%	61.12%	29.58%	7.28	7.89
11	26.80%	59.10%	14.10%	0.94	1.35	12.71%	57.77%	29.52%	2.37	2.69
12	38.84%	51.25%	9.91%	0.60	1.80	8.94%	63.69%	27.37%	3.32	4.01
13	9.92%	52.03%	38.05%	2.17	2.74	3.54%	30.90%	65.57%	9.53	9.90
Average	26.92%	51.14%	21.94%	1.40	1.96	7.68%	46.24%	46.08%	5.69	6.15

^a^ After normalization.

Since each hair FU extracted has different number of hairs, we divide these hair FUs into one‐haired FUs, two‐haired FUs, and three‐haired FUs according to the number of hair shafts contained in each hair FU. Although some of the hair would be accidentally cut or injured during the extraction process, for hair FUs containing multiple hairs, cutting some of the hair in the hair FUs did not affect the implantation and utilization of the whole hair FUs. If all hairs in a hair follicle were cut, the hair follicle cannot be used for hair transplantation and is recorded as discarded hair follicle. Our calculation of discard rate thus reflects the damage of hair FUs. On the premise of extracting the same number of hair follicles, the more discarded hair follicles, the higher the discard rate, and the fewer hair follicles that can be used for implantation in the recipient area, indicating lower hair extracting efficiency.

**FIGURE 2:**
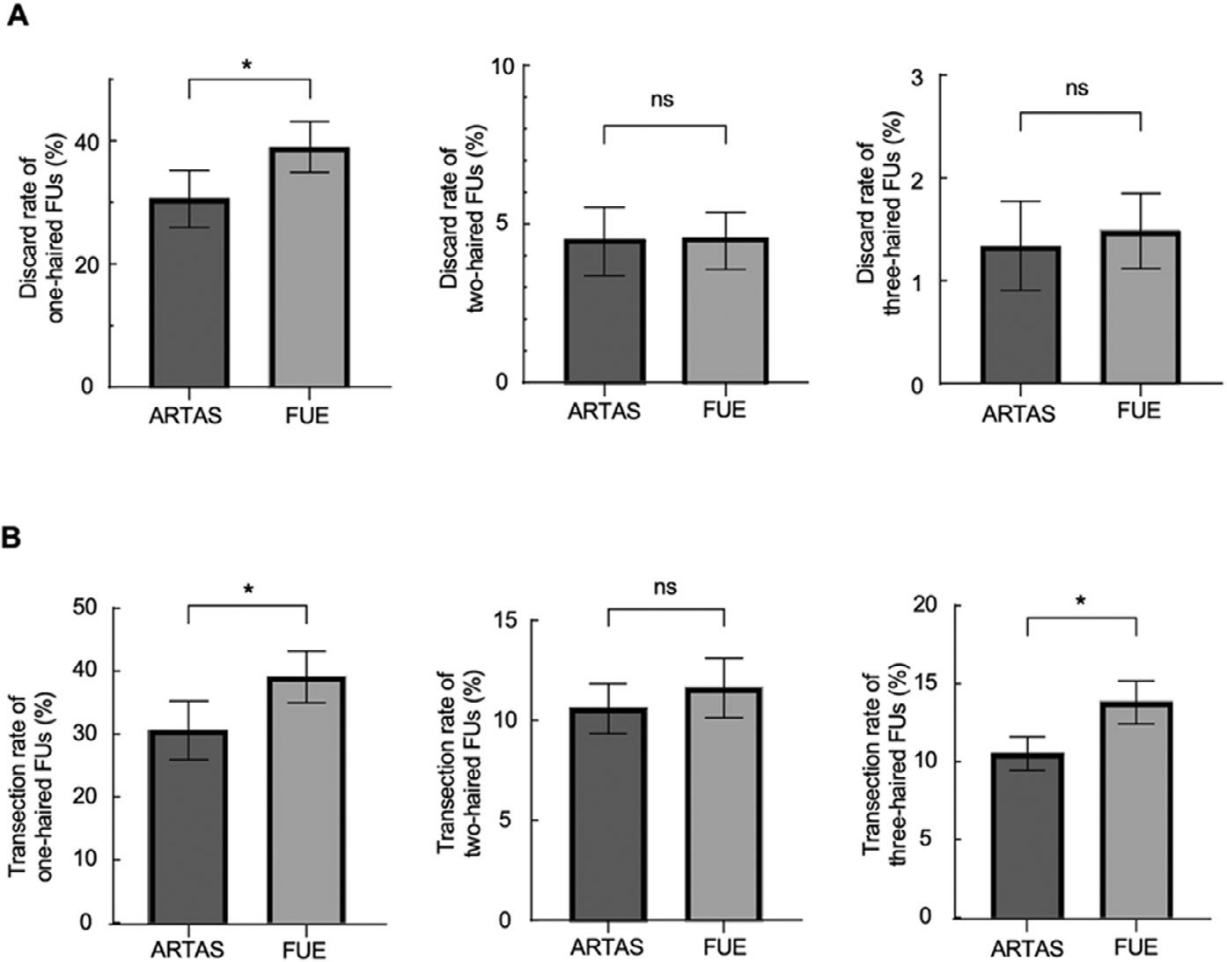
(A) Discard rate and (B) transection rate of different types of FUs. “*” stands for statistical difference (*p* < 0.05), while ns stands for no statistical difference.

According to Figure 2A, the discard rate of one‐haired FUs of the ARTAS side was significantly lower than that of the FUE side, 30.15% and 38.85%, respectively. The discard rate of two‐haired FUs of the ARTAS side was lower than that of the FUE side (5.18% vs. 5.25%), and there was no statistical difference between the two groups (*p* > 0.05). The discard rate of three‐haired FUs of the ARTAS side was lower than that of the FUE side (1.34% vs. 1.49%), and there was no statistical difference between the two groups (*p* > 0.05).

Hair transection occurred during hair follicle extraction. The transection rate was obtained by dividing the number of transected hairs by the number of all hairs. It can be shown that when the number of extracted hairs was the same, the more transected hairs, the fewer hairs that can be implanted in the receiving area, and the lower the extraction efficiency. According to Figure 2B, we classified the extracted hair follicles and calculated the transection rates of different types of hair FUs. The transection rate of one‐haired FUs of the ARTAS side was significantly lower than that of the FUE side, 30.15% and 38.85%, respectively. The transection rate of two‐haired FUs of the ARTAS side was lower than that of the FUE side (11.21% vs. 13.72%), and there was no statistical difference between the two groups (*p* > 0.05). The transection rate of three‐haired FUs of the ARTAS side was significantly lower than that of the FUE side, 10.50% and 13.80%, respectively.

**FIGURE 3:**
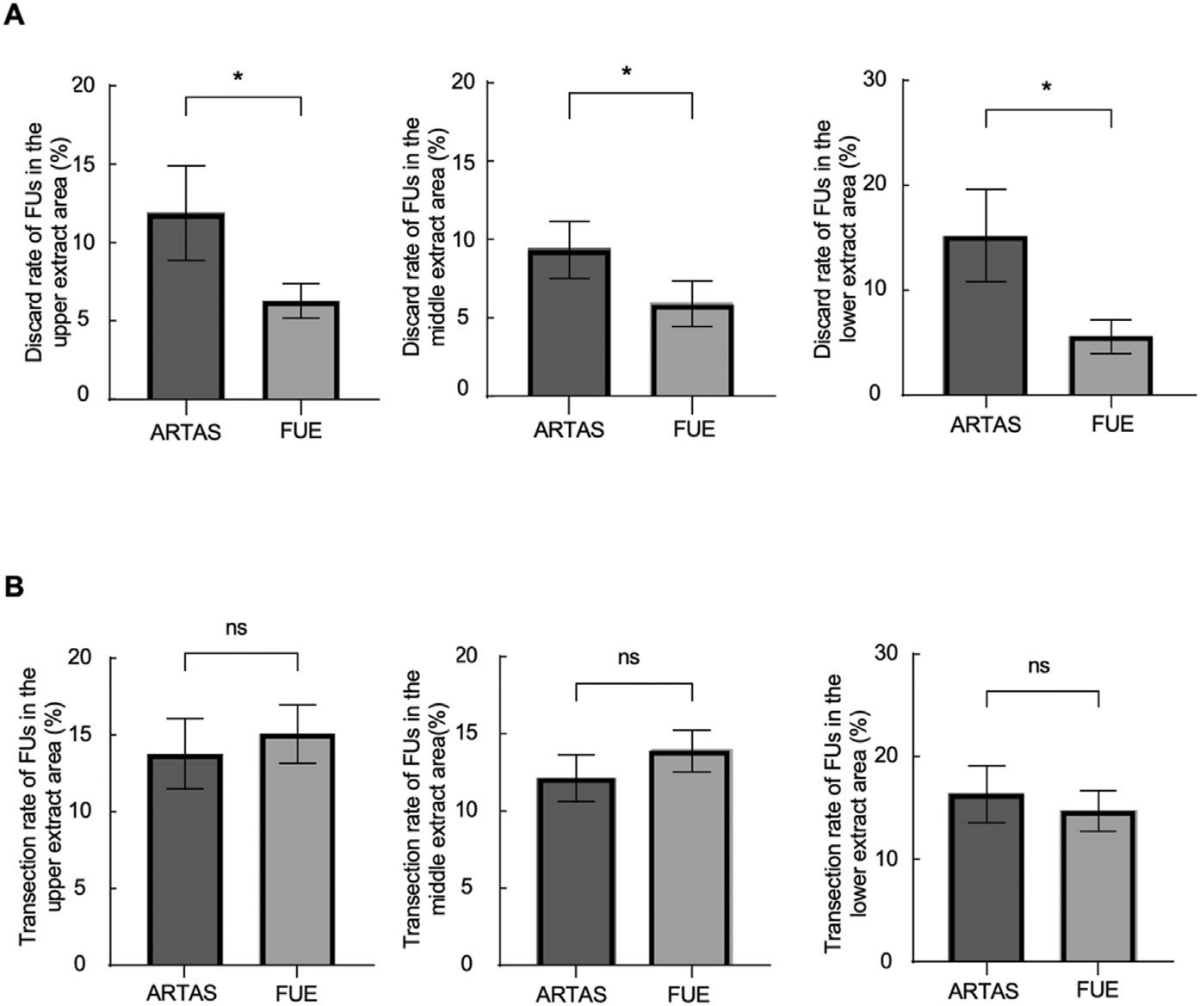
Discard rate and transection rate of FUs from different extract areas in ARTAS and FUE. “*” stands for statistical difference (*p* < 0.05), while ns stands for no statistical difference.

In order to further compare the performance of ARTAS and FUE in different hair follicle extraction areas, we divided the surgical area into upper extract area, middle extract area and lower extract area, and compared the transection rate and discard rate of these areas (Figure 3). The most upper boundary was defined the area between 3 and 4 cm below the hairline and the lowest boundary was 3 cm above the occipital hairline. The area in the middle were divided evenly into four, with the top and bottom zones taking up 25% each. The remaining 50% most appropriate for extraction was the middle zone. In the upper extract area, the discard rate of ARTAS was significantly higher than that of the FUE side, 11.88% versus and 6.27%, respectively (Figure 3A). In the middle extract area, the discard rate of ARTAS was significantly higher than that of the FUE side, which was 9.34% and 5.91%, respectively (Figure 3A). In the lower extract area, according to Figure 3A, the discard rate of ARTAS was significantly higher than that of the FUE side, 12.83% and 5.59%, respectively. In the upper extract area, the transection rate of ARTAS was lower than that of the FUE side (13.75% vs. 15.05%) (Figure 3B). In the middle extract area, the transection rate of ARTAS was lower than that of the FUE side (12.12% vs. 13.88%) (Figure 3B). In the lower extract area, the transection rate of ARTAS was higher than that of the FUE side (14.94% vs. 14.74%) (Figure 3B). But these differences were not statistically significant.

**FIGURE 4:**
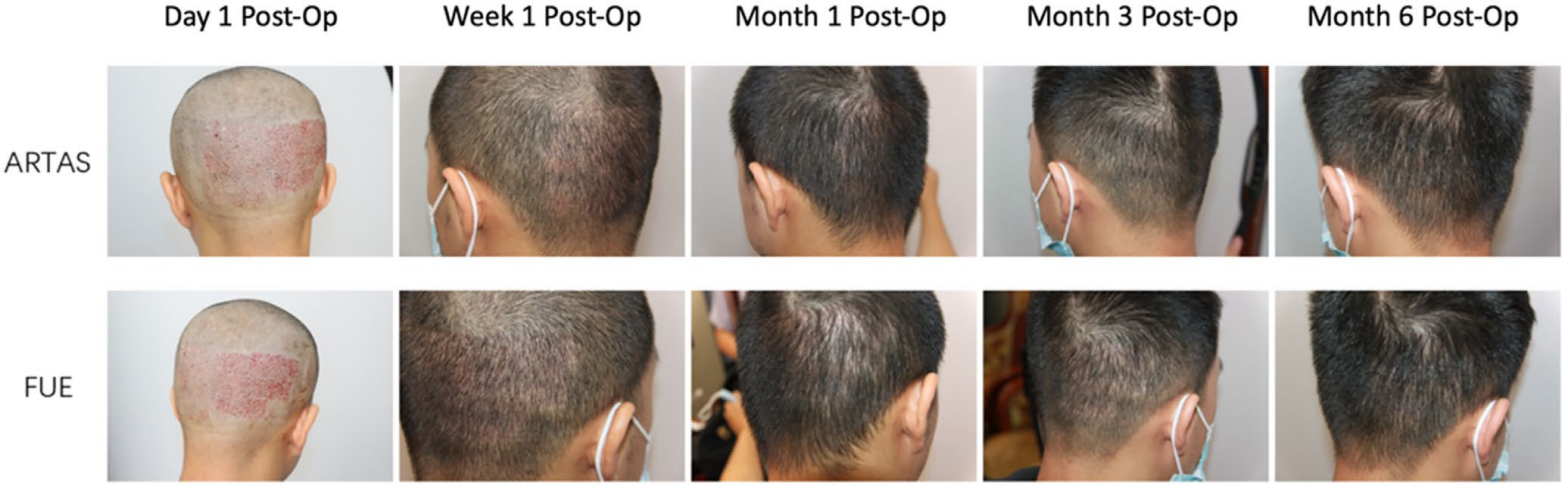
Postoperative recovery of donor area scalp in ARTAS and FUE.

**FIGURE 5:**
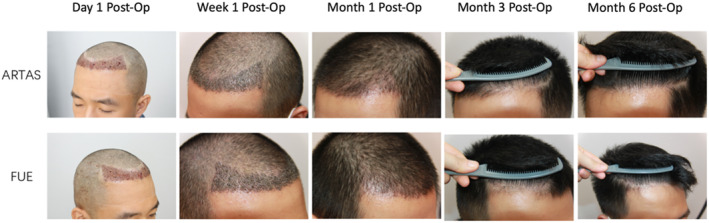
Postoperative recovery of recipient area scalp in ARTAS and FUE.

The follow‐up period was 12 months, with data showing no significant difference in hair regrowth and patient satisfaction between ARTAS and FUE (Figures 4 and 5).

### 3.3 Safety

During the whole treatment and follow‐up, no significant side effects or complications were detected during or after the surgery. During ARTAS or FUE, no cases of infection or excessive scar formation were seen, and no patients reported severe pain, suggesting that the two operation methods were safe.

## 4 Discussion

AGA is a multifactorial disease caused by the joint action of gene, endocrine, and aging [7]. Androgen receptor sensitivity is the main factor of the disease. At present, it is generally believed that the pathogenesis of AGA is closely related to DHT, which causes hair follicle miniaturization, progressive shortening of hair follicle growth period, and eventual hair loss [8]. The hair loss caused by AGA does not directly threaten life, but brings negative emotions that may lead to depression [9]. Therefore, the treatment of AGA is in great demand, which constitutes a multibillion‐dollar industry [10].

The goal of treatments is to increase the coverage of hair or delay the further thinning of hair [11]. Although treatments including finasteride and 2% or 5% minoxidil solution have the best effect when initiated early, any benefit may be lost if the treatment is stopped [12]. In addition, if complete atrophy of hair follicles has occurred, the effect of drug treatments may be poor. Hair transplantation is the only permanent successful operation at present [12]. Among the two methods of hair transplantation, FUE has become the current mainstream operation over FUT due to its advantages of small injury, less visible scars, fast recovery, and even less impact on daily life. However, the challenges of this technology include the increased risk of hair follicle transection and the fatigue caused to surgeons when the number of hair FUs increase. The emergence of robotic technology is an important progress in hair transplantation. The FDA certified ARTAS robotic system (Restoration Robotics, Inc, San Jose, CA) is a relatively well‐known device on the market.

Rose [13] summarized that the advantages of ARTAS system were small damage, fast healing, and no linear scar. Using ARTAS system, the learning time cost of hair transplant doctors was greatly reduced, the workload was reduced, and the hair transection was relatively lower. Some clinical studies on ARTAS have been carried out abroad. The study of Avram and Watkins [6] involved 20 cases of hair transplantation with ARTAS system. The results showed that the average transection rate of hair follicle extraction with ARTAS was 6.6%. As for Asian patients, Shin et al. [14] analyzed 22 patients in South Korea who used ARTAS system for hair transplantation and found that the transection rate was 4.91% ± 2.9%. The extracted hair FUs were classified according to the number of hair shafts. The hair FUs containing two hairs accounted for 44.1%, and the hair FUs containing three hairs accounted for 31.9%. The study concluded that the ARTAS system can not only harvest single‐haired FUs effectively, but also harvest FUs with multiple hairs.

In 2016, Bernstein and Wolfeld [15] studied 24 cases with ARTAS system, and compared random FU harvesting with selective graft harvesting under human intervention. The results showed that much more hair FUs with multiple hair shafts could be obtained when selective graft harvesting was conducted under human intervention. When the same number of hair FUs were extracted, the postoperative effect of selective graft harvesting was better. For the extraction of more than 2000 hair FUs, robotic hair transplantation technology can still be used. Pereira et al. [16] studied 157 patients who used the ARTAS system for hair transplantation and found that 67% of the patients had more than 2000 hair FUs transplanted. Compared with the clinical photos taken before and after the transplantation, it was found that the treatment effect was good, which showed that the ARTAS system had high feasibility and safety in the hair transplantation involving a large number of hair FUs. Although these studies illustrate the advantages of robotic systems, there is a lack of comparative research with traditional FU extraction methods. Therefore, this study was carried out to compare ARTAS technology with FUE and further explore the improvement of ARTAS in hair transplantation.

In the present study, the transection rate of different types of hair FUs extracted by ARTAS was lower than that of FUE, seeing a significant reduction of transection rate of one‐haired FUs. The total transection rate was also lower than that of FUE side, which indicates that the transection of hair FUs extracted by ARTAS is less likely to occur. The main reason is that ARTAS has a high‐precision dual‐camera imaging system, which can evaluate the number and distribution of hair follicles in real time, accurately analyze the direction and angle of hair follicles, and automatically select high‐quality hair follicles. In addition, ARTAS adopts a double‐needle system (the inner needle is sharp, which can penetrate the epidermis, and the outer needle is blunt, which can rotate and cut to separate hair follicles), which can reduce the transection rate of hair follicles and assist in the evaluation of puncture depth. The hair transplant surgeon can adjust the depth and puncture angle of the inner and outer needles at any time.

After observing the discard rate of different types of hair FUs, it is not difficult to find that the discard rate of one‐haired FUs was more than 30%, which was much higher than that of two‐haired and three‐haired FUs, yielding a 20‐fold difference. The reason may be that when one hair in the one‐haired FUs was damaged, the whole hair follicle would be discarded. For a three‐haired FU, even if one or two hairs were damaged, the whole hair follicle would not be unusable.

The overall higher discard rate for ARTAS compared to FUE, despite lower rates for individual hair FUs, can be attributed to the weighted average calculation of the total discard rate. Since ARTAS extracts a higher proportion of one‐haired FUs (nearly 30% compared to FUE's 7%), and these have a discard rate over 10%, the overall discard rate for ARTAS rises to 10.71%, which is above the typical 5%–8% reported for FUE [17]. This disparity is due to the specific conditions and parameters of our study, emphasizing that the more one‐haired FUs extracted, the closer the total discard rate aligns with their individual rates, subsequently lowering the overall yield for ARTAS. After normalizing the data concerning discard and transection rates however, ARTAS yielded much better results (Table 5). Further study initiated with set portions of hair count in follicles in its design should be conducted for more in‐depth investigation.

In the middle extract area, the transection rate of FUs on the ARTAS side was lower than that on the FUE side (12.12% vs. 13.88%), and the difference of discard rate between the ARTAS side and the FUE side was the smallest (9.34% on the ARTAS side, 5.91% on the FUE side, the difference between the two was 3.43%). In addition to the above discussion on the discard rate of ARTAS, the hair quality in the upper and lower extract areas of the scalp was relatively poor compared with that in the middle extract area, which was mainly reflected in the relatively small diameter of hair and the relatively large proportion of one‐haired hair FUs.

We acknowledge the limitation of a small sample size and its potential impact on the study's findings. Future studies with larger sample sizes are needed to confirm these results. It can be expected that after optimizing the algorithm and parameters in the future, ARTAS will have even better performance in extracting hair FUs with multiple hairs, and can extract more hair follicles with complete structure. In addition, using ARTAS in the relatively safe extraction area (middle extract area) may improve the efficiency of extraction.

## 5 Conclusion

In conclusion, the current iteration of ARTAS can achieve the same patient satisfaction (efficacy) to FUE done by experienced surgeons, and is just as safe. While the yield rate is lower in ARTAS (not statistically significant), the transection rate is lower. Clinically, this means that follicles once successfully harvested by ARTAS have a higher chance to be of higher quality compared to FUE counterparts.

## Ethics Statement

This single‐center, prospective, randomized, single‐blinded, controlled, split‐scalp pilot study was conducted at Huashan Hospital, Fudan University between April 2020 and June 2021 (identifier: ChiCTR2000030220). The study protocol was approved by the Huashan Institutional Review Board and independent ethics committees and conformed to the ethical guidelines of the Declaration of Helsinki and Good Clinical Practice. All patients provided written informed consent and agreed to the use and analysis of their data, prior to any study‐related procedures. The study protocol was approved by the Huashan Institutional Review Board and independent ethics committees and conformed to the ethical guidelines of the Declaration of Helsinki and Good Clinical Practice. All patients provided written informed consent and agreed to the use and analysis of their data, prior to any study‐related procedures.

## Consent

All authors consent to publishing this article.

## Conflicts of Interest

The authors declare no conflicts of interest.

## Data Availability

The data that support the findings of this study are available from the corresponding author upon reasonable request.
